# T Cells and Adoptive Immunotherapy: Recent Developments and Future Prospects in Gastrointestinal Oncology

**DOI:** 10.1155/2011/320571

**Published:** 2011-11-03

**Authors:** Amedeo Amedei, Elena Niccolai, Mario M. D'Elios

**Affiliations:** ^1^Department of Internal Medicine, University of Florence, Viale Morgagni 85, 50134 Florence, Italy; ^2^Dipartimento Biomedicina, Azienda Ospedaliera Universitaria Careggi, Viale Morgagni 85, 50134 Florence, Italy

## Abstract

Gastrointestinal oncology is one of the foremost causes of death: the gastric cancer accounts for 10.4% of cancer deaths worldwide, the pancreatic cancer for 6%, and finally, the colorectal cancer for 9% of all cancer-related deaths. For all these gastrointestinal cancers, surgical tumor resection remains the primary curative treatment, but the overall 5-year survival rate remains poor, ranging between 20–25%; the addition of combined modality strategies (pre- or postoperative chemoradiotherapy or perioperative chemotherapy) results in 5-year survival rates of only 30–35%. Therefore, many investigators believe that the potential for making significant progress lies on understanding and exploiting the molecular biology of gastrointestinal tumors to investigate new therapeutic strategies such as specific immunotherapy. In this paper we will focus on recent knowledge concerning the role of T cells and the use of T adoptive immunotherapy in the treatment of gastrointestinal cancers.

## 1. Introduction

Gastrointestinal oncology is one of the foremost causes of death; regarding the gastric cancer (GC) the American Cancer Society estimated one million new cases, nearly 70% of them in developing countries, and about 800,000 deaths [1]; instead the pancreatic cancer (PC) is the fourth leading cause of cancer deaths among men and women, being responsible for 6% of all cancer-related deaths [2], and finally, the colorectal cancer (CRC) accounted for 9% of all cancer deaths (49, 920) in 2009 [3]. 

For all these gastrointestinal cancers, surgical tumor resection remains the primary curative treatment but the overall 5-year survival rate remains poor, ranging between 20–25% [4–6]. The addition of combined modality strategies (pre- or postoperative chemoradiotherapy or perioperative chemotherapy) results in 5-year survival rates of only 30–35% [7–9]. 

Therefore, many investigators believe that the potential for making significant progress lie on understanding and exploiting the molecular biology of gastrointestinal tumors to investigate new therapeutic strategies such as gene therapy [10] and especially specific immunotherapy [11–13].

Evidence from different analysis suggests a key role of the immune system in counterattack of cancer progression: tumors are 100 times more likely to occur in people who take immunosuppressive medications than in people with normal immune function [14], and, in opposition, heightened anti-tumor activity of the immune system has been suggested in many reports of spontaneous cancer regression [15]. Also, a positive correlation between tumor-infiltrating lymphocytes and patients' survival has been observed [16]; moreover tumor-specific T-cell responses have been found in patients with a variety type of tumors [17]. 

**Figure 1:**
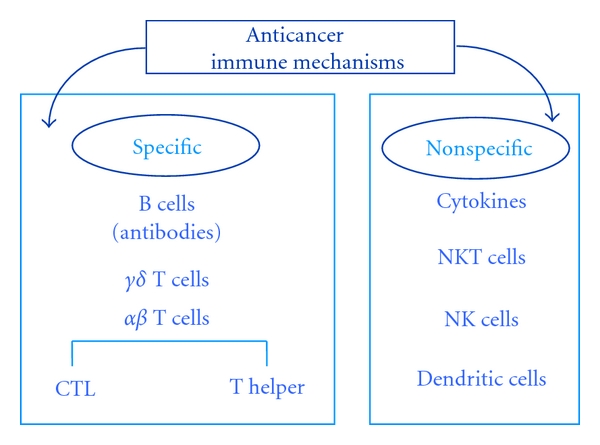
Innate and adaptive immune defence against cancer cells.

Immune defence against tumor is mediated through antigen-specific and nonspecific immune mechanisms (macrophage and NK cell lineage and soluble factors such as cytokines). The operational, instead, of the antigen-specific immune system is based on a division of tasks between T cells and B cells (Figure 1). 

Various reagents (vaccines, infusion of T cells, or cytokines) can stimulate the immune system essentially through two mechanisms: (1) stimulation of the antitumor response, either by increasing the number of effector cells or by producing soluble mediators (e.g., cytokines); (2) alteration of tumor cells to increase their immunogenicity and susceptibility to immunological defences. However, the cancer cells have developed a number of different strategies to escape immune surveillance such as loss of tumor antigen expression, MHC downregulation, expression of Fas-L that can induce apoptosis in activated T cells, secretion of cytokines such as IL-10 (Interleukin-10) or TGF-*β* (Tumor grow factor-*β*), or generation of regulatory T (Treg) cells [18]. 

The requirement for an immune-based strategy in opposition to cancer is the induction of an effective tumor-specific immunity in order to break immunological tolerance to the tumor and generate antitumor immunity. To achieve this goal, several strategies as in preclinical models as in clinical trials are currently being investigated. 

In this paper we will focus on recent knowledge concerning the role of T cells and the use of T adoptive immunotherapy in the treatment of gastrointestinal cancers.

## 2. Pancreatic Cancer 

### 2.1. In Human and Animal Model T-Cell Response

Over the past 30 years, a large body of data has been accumulated showing that cancer patients generate B and T cells specific to antigens expressed on autologous pancreatic tumor cells [19–25]. PC expresses a variety of cancer-associated antigens that can potentially be recognized by T cells [26, 27]. Recent studies demonstrated that functionally competent CD4^+^ and CD8^+^ T cells with specificity for cancer antigens are spontaneously induced in the bone marrow of all PC patients [27, 28]. Moreover, in approximately 50% of the patients, these tumor-specific T cells are also present in the blood. Upon specific stimulation they mainly secrete the type 1 cytokine IFN-*γ*, which is typical of cytotoxic immune responses. The high incidence of spontaneous T-cell reactivity versus PC is in contrast to observations from numerous other cancer entities that induced cancer-reactive T cells only in 25–60% of the patients [29–31].

**Figure 2:**
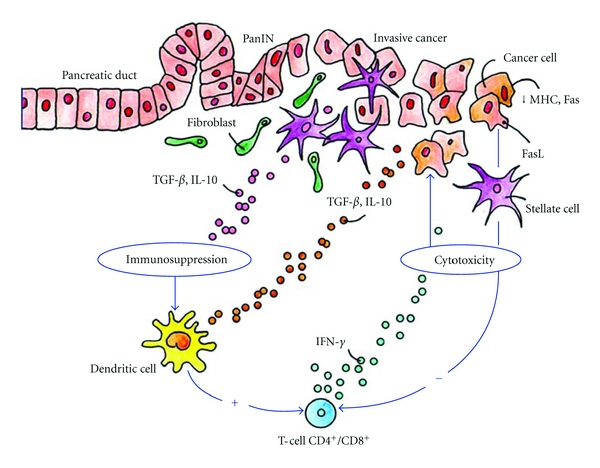
Pancreatic cancer microenvironment: interactions of immune cells with the cancer cells. Yellow: products of stellate cells; green: T-cell derived cytokines; grey: cancer cell-derived factors.

T-cell responses are regulated by dendritic cells (DCs), which constantly take up antigens in all tissues and upon in situ activation, stimulate naive T cells. While type I interferons, heat shock proteins, and extracellular matrix degradation products may induce DC activation in cancer tissues, immune-suppressive cytokines (IL-10/TGF-*β*) inhibit DC activation, and in PC the latter are produced at high concentrations by cancer-induced pancreatic stellate cells, cancer-infiltrating macrophages and mast cells [32], or Tregs [33]. Through recruitment and activation of stroma cell populations, PC generates a predominantly immune-suppressive microenvironment (Figure 2).

The regular induction of T-cell responses in the bone marrow of PC patients is thus intriguing. 

The bone marrow is a site of T-cell priming against blood-borne antigens [34]. It can collect soluble cancer antigens released into the blood from necrotic cancer areas. Here, these are incorporated and presented by bone marrow-resident DCs in an immune-stimulatory environment. In addition, disseminated neoplastic cells detectable in many patients represent a local source of cancer antigens [28]. 

PC is frequently diagnosed at late stages. In this situation, large antigen amounts may reach the bone marrow. This might explain the comparably high incidence of T-cell responses in PC despite a predominantly immune-suppressive environment in the primary cancer. Once stimulated, T cells differentiate into effector T cells and enter the blood. Since cancer-reactive T cells have been found in the blood of many PC patients, these cells may infiltrate pancreatic carcinomas.

In one study, cancer-reactive CD8^+^ T cells specifically lysed autologous PC cells *in vitro* and delayed progression of xenotransplanted, autologous carcinomas [27]. Accordingly, increased numbers of cancer-infiltrating CD4^+^ and CD8^+^ T cells correlated well with improved prognosis of PC patients [35].

These findings point to a potential implication of cancer- specific T cells during cancer progression, but PC cells successfully employ various mechanisms to evade immune surveillance (Figure 2): (a) the downregulation of MHC molecules and of fas receptor, rendering neoplastic cells more resistant to recognition and cytolysis by activated effector T cells [27], (b) the recruitment and local maintenance of Tregs [36] that inhibit effector T-cell activation and function, (c) the secretion of IL-10 and TGF-*β*, additionally reducing local T-cell activity [27, 37], (d) the inactivation of cancer-infiltrating T cells as shown by a severe loss of CD3 zeta, [37] and (e) the expression of fas ligand on neoplastic cells, inducing apoptosis in cancer-infiltrating effector T cells [38].

Thus, PC is not characterized by a lack of specific T-cell immunity but by a potent barrier established by complex cancer-stroma interactions that inhibit T-cell activity in situ; for this purpose is most explanatory the recent results obtained by De Monte et al. [39]; they showed that thymic stromal lymphopoietin (TSLP), which favors Th2 cell polarization through myeloid DC conditioning, was secreted by cancer-associated fibroblasts (CAFs) after activation with tumor-derived TNF-*α* and IL-1*β*. Also the authors found that the ratio of GATA-3^+^(Th2)/T-bet^+^ (Th1) tumor-infiltrating T cells is an independent predictive marker of patient survival. Patients surgically treated for stage IB/III disease with a ratio inferior to the median value had a statistically significant prolonged overall survival, implying an active role for Th2 responses in disease progression. 

In addition, in a mouse model in which an activating K-Ras mutation is expressed in the pancreas, preinvasive pancreatic lesions are characterized by the infiltration of immune suppressor cells rather than immune effector cells, suggesting that tumor immunity may be blocked from the inception of PC development [40]. 

All mice with the K-Ras mutation develop pancreatic adenocarcinoma and eventually die of disease. Finally, the finding that antagonism of negative T-cell regulators, such as cytotoxic T-lymphocyte-associated (CTLA) protein-4 and B- and T-lymphocyte attenuator (BTLA), can augment the antitumor immune response confirms that patients mount an immune-specific response to their tumor [41, 42]. Despite mounting evidence that an antitumor immune response is elicited in cancer patients, this response is ineffective and does not result in the tumor eradication, and a better understanding of the mechanisms underlying these interactions is required to develop future therapeutic strategies to employ the patient's own T-cell arsenal for efficient cancer control.

### 2.2. T-Cell Immunotherapy of Pancreatic Cancer

The history of vaccine trials in pancreatic cancer targeting a defined PC antigen started with the publication of a pilot study of mutant ras peptide vaccines tailored to represent the K-RAS mutations identified in biopsies from the patients with cancer [43] In this trial, immune responses specific for individual ras mutations were obtained in 2 of the 5 patients enrolled; in addition, both patients had a relatively long survival (11 and 8 months). These data shown that: (a) patients with metastatic PC were immunocompetent, (b) mutant ras vaccines were immunogenic, and (c) immune responses were correlated with survival. Furthermore, the treatment was well tolerated as no adverse effects were observed. A fine evaluation of the immune responses in these two patients [44] highlighted that peptide vaccination with a single mutant p21-ras-derived peptide induced CD4^+^ and CD8^+^ specific for nested epitopes, including the Gly*/*Val substitution at codon 12 and that both these T-cell subsets specifically recognize tumour cells owning to the corresponding K-ras mutation. Encouraged by these results, a second trial was performed, using intradermal vaccination of mutant ras peptides with GMCSF (Granulocyte-macrophage colony-stimulating factor) as an adjuvant [45]. 48 patients (10 surgically resected and 38 with advanced disease) were treated on an outpatient basis. Peptide-specific immunity was induced in 25 of 43 (58%) evaluable patients, indicating that the protocol used is very potent and able to elicit immune responses even in patients with end-stage disease. This study also demonstrated a strong association between immune responses and prolonged survival. Patients with advanced cancer and with immune response to the peptide vaccine showed prolonged survival from the start of treatment compared to nonresponders (median survival 148 days versus 61 days). Furthermore, the study proved long-term memory in numerous patients and entry of vaccine-specific T cells into the tumour mass.

In recent years, much work has focused on adoptive tumor immunotherapy in which the T cells of cancer patient are expanded and reinfused into the patient. 

One method results in the selective expansion of T cells endogenously expressing TCRs specific for the tumor antigen of interest [46]. In a clinical study, MUC-1-specific autologous T cells, isolated from patient PBMCs (peripheral blood mononuclear cells), were expanded by incubation with a MUC-1-presenting cell line prior to administration to PC patients. The mean survival time for unresectable patients in this study was 5 months [47]. However, patients with resectable pancreatic cancer had 1-, 2- and 3-year survival rates of 83.3, 32.4, and 19.4%, respectively, and a mean survival time of 17.8 months. In a similar study, the same group isolated adherent cells from patient PBMCs to generate mature DCs that were then pulsed with MUC-1 peptide. The pulsed DCs were administered, along with autologous expanded MUC-1-specific T cells, to patients with unresectable or recurrent pancreatic cancer. Remarkably, a complete response was observed in one patient with lung metastases, and the mean survival time of the whole group was 9.8 months, suggesting that the addition of pulsed DCs may have improved the outcome [48].

A key role in future immunotherapeutic treatment of PC patients seems to be for the novel antigen PC-associated *α*-enolase (ENOA), a metabolic enzyme involved in the synthesis of pyruvate. In tumor cells, ENOA is upregulated and supports anaerobic proliferation (Warburg effect); also, it is expressed at the cell surface, where it promotes cancer invasion. ENOA is upregulated in different tumors, including brain, breast, cervix, colon, gastric, kidney, lung ovary, and especially pancreas [49].

In pancreatic cancer, ENOA elicits a CD4^+^ and CD8^+^ T-cell response both *in vitro* and *in vivo* [49]. Anti-MHC class I antibodies inhibited the cytotoxic activity of ENOA-stimulated CD8^+^ T lymphocytes against PC cells, but no MHC class I restricted peptide of ENOA has been identified so far. Moreover, in pancreatic ductal adenocarcinoma patients, production of anti-ENOA Immunoglobulin-G (IgG) is correlated with the ability of T cells to be activated in response to the protein [49], thus confirming the induction of a T- and B-cell integrated antitumor activation against ENOA. In oral squamous cell carcinoma, an HLA-DR8-restricted peptide (amino acid residues 321–336) of human ENOA recognized by CD4^+^ T cells and able to confer cytotoxic susceptibility has been identified [50, 51]. 

Most importantly, clinical correlations [52–54] propose ENOA as a novel target for cancer immunotherapy, in particular in pancreatic cancer, where pancreas-specific Ser 419 phosphorylated ENOA is upregulated and also induces the production of autoantibodies with diagnostic and prognostic value [49].

## 3. Gastric Cancer 

### 3.1. Gastric Cancer-Infiltrating T Cells 

Although the GC etiology has been completely obscure for many decades, several considerable advances in the knowledge of the carcinogenesis and development of gastric cancer have been made in the present era. First, it is well known that *Helicobacter pylori *(*H. pylori*) infection is associated with the GC carcinogenesis, suggesting that chronic inflammation may be implicated in the development of intestinal metaplasia and mutations in oncogenes that precede the GC development; indeed, the International Agency for Research on Cancer classified *H. pylori *as a class I human carcinogen in 1994 [55]. Second, the long-suspected influence of genetic susceptibility has been elucidated, and several polymorphisms of inflammatory cytokine genes have been implicated as risk factors for gastric cancer [56–60].

Although immune cells constitute an additional and prominent component of the host response to cancer, their participation in tumor pathogenesis remains unclear. In the tumor microenvironment, there is a delicate balance between antitumor immunity and tumor-originated proinflammatory activity, which weakens antitumor immunity [61–63].

It has been shown that the infiltrating grade of CD3^+^ tumor-infiltrating lymphocytes (TILs) was correlated with a favorable outcome in patients with several types of cancer, including gastric cancer [64]. Thus, it is imperative to understand immunoregulation in gastric cancer, in order to develop novel treatment strategies or improve the efficacy of standard therapies.

The first evidence of correlation between T-cell response and GC was the study of Ren et al. [65] that reported a shift from Th1 to Th2 pattern of cytokine secretion in gastric cancer and has suggested that this may be a critical factor in promoting growth of neoplastic cells. However, our data [66] of tumor-infiltrating and perilesional *H. pylori*-specific T cells failed to confirm such a Th1-Th2 shift. Rather, the major difference between the gastric T-cell clones from uncomplicated chronic gastritis and those from gastric cancer was the degree of expression of cytolytic activity. Indeed, in all patients studied, virtually all the *H. pylori*-specific CD4^+^ clones derived from gastric tumors or perilesional mucosa consistently expressed perforin-mediated cytolytic potential and Fas-Fas ligand-mediated proapoptotic activity against target cells.

Most recently, Maruyama et al. [67] investigated the distribution of Th17 (T helper 17) cells in relation to Treg as in the TILs as in peripheral blood of GC patients. They showed that in TILs from patients with early disease, the frequency of Th17 cells was significantly higher than that in the normal gastric mucosa (23.7 ± 8.9 versus 4.5 ± 3.1%). Besides, in TILs from patients with advanced disease (*n* = 28), the frequency of Th17 cells was also significantly higher, but lower compared to early disease, than that in the normal gastric mucosa (15.1 ± 6.2 versus 4.0 ± 2.0%). When the ratio of Th17/Treg in TILs was evaluated in individual cases, it was more markedly increased in early than in advanced disease. 

In summary, the accumulation of Th17 cells as well as Tregs in the tumor microenvironment of gastric cancer occurred in early disease, and then the infiltration of Th17 cells gradually decreased according to the disease progression, in contrast to increased Tregs.

### 3.2. T-Cell-Based Antigastric Cancer Treatments

There are different types of T-cell-based anticancer therapy approaches, using (a) CTL, (b) TILs, or (c) Engineered T cells. 

Improved CTL cell culture technology has permitted the first clinical tests for adoptive transfer of CTLs, and this technique [68] seems to result in substantial activity in patients with melanoma; CTLs were used to treat patients with metastatic melanoma, and 8 out of 20 patients had anti-tumor immune responses [68]. These results were confirmed in an independent trial in which engraftment of the CTLs, as measured by an elevated frequency of circulating T cells able to bind tetramers loaded with MART-1 peptides, was detectable up to two weeks after T-cell transfer in all patients [69].

Recently, Kim et al. [70] evaluated the antitumor activity of *ex vivo *expanded T cells against the human gastric cancer. For this purpose, human peripheral blood mononuclear cells were cultured with IL-2-containing medium in anti-CD3 antibody-coated flasks for 5 days, followed by incubation in IL-2-containing medium for 9 days. The resulting populations were mostly CD3^+^ T cells [97%] and comprised 1% CD3^−^CD56^+^, 36% CD3^+^CD56^+^, 11% CD4^+^, and 80% CD8^+^. This heterogeneous cell population was also called cytokine-induced killer (CIK) cells. CIK cells strongly produced IFN-*γ*, moderately TNF-*α*, but not IL-2 and IL-4. At an effector-target cell ratio of 30 : 1, CIK cells destroyed 58% of MKN74 human gastric cancer cells, as measured by the 51Cr-release assay. In addition, CIK cells at doses of 3 and 10 million cells per mouse inhibited 58% and 78% of MKN74 tumor growth in nude mouse xenograft assays, respectively. This study suggests that CIK cells may be used as an adoptive immunotherapy for GC patients.

The adoptive GC immunotherapy with CIK cells has been also reported in preclinical and clinical studies [71]. MHC-I restricted CTLs from GC patients recognize tumor-associated antigen and react specifically against self-tumor cells [72, 73]. One tumor-specific antigen, MG7-antigen, shows great potential for predicting early cancer as well as for inducing immune responses to GC [74, 75]. Using HLA-A-matched allogeneic gastric cancer cells to induce tumor-specific CTLs appears to be an alternative immunotherapy option for gastric cancer [76]. 

Also, CIK cells in combination with chemotherapy showed benefits for patients who suffer from advanced gastric cancers [77, 78]. The serum levels of the tumor markers were significantly decreased, the host immune function was increased, and the short-term curative effect, as well as the quality of life, was improved in patients treated by chemotherapy plus CIK cells compared to those in patients treated by chemotherapy alone. CIK cells killed MGC-803 GC cells by inducing apoptosis in the early stage and by inducing necrosis in the late stage through downregulation of p53, c-myc, and bcl-2 and upregulation of bax [79].

In summary, despite the introduction of immune cell-based immunotherapy, the paucity of preclinical and clinical studies has limited the broad application of immunotherapy for the treatment of GC patients with gastric cancers. Here, preclinical evidence proved that CIK cell immunotherapy can be used in patients with gastric cancer. 

**Figure 3:**
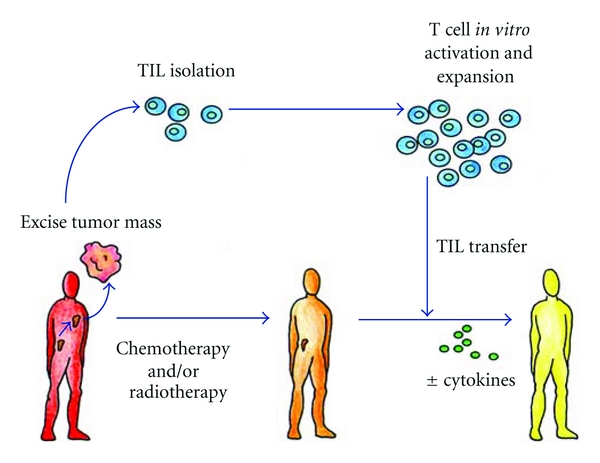
Scheme of adoptive autologous TILs transfer. T-infiltrating lymphocytes can be isolated from resected surgical samples and expanded *in vitro* for adoptive transfer after lymphodepleting chemotherapy. Most adoptive transfer therapy approaches using TILs have involved the use of IL-2 infusion following T-cell transfer in order to select tumor-specific T cells.

Adoptive transfer therapy with TILs requires the isolation of T cells from neoplastic biopsies or surgical tissue and the selection of tumor-specific T cells *ex vivo* (Figure 3). The adoptive transfer of TILs has been promising in preclinical models [80], but clinical experiences were almost uniformly disappointing [81, 82]. 

Technical difficulties in producing tumor-specific T cells currently represent a barrier to randomized clinical trials. Only 30%–40% of the biopsies yield satisfactory T-cell populations, and the whole process requires about 6 weeks before the T cells would be ready for infusion [83]. Furthermore, nearly all clinical experiences with TILs have been done in patients with melanoma, because of the easy surgical availability of the tumor tissue. However, should technical limitations of current tissue culture approaches be overcome, recent studies indicate that the presence of TILs positively correlates with patients survival in ovarian and colorectal cancer [84, 85], thus prompting the use of this protocol for other commonly encountered epithelial neoplasias. Recently we have [11] analyzed the functional properties of the T-cell response to different antigen peptides related to GC in patients with gastric adenocarcinoma. To this purpose, we have cloned and characterized TILs isolated from the neoplastic gastric tissue samples. A T-cell response specific to different peptides of gastric cancer antigens tested was documented in 17 out of 20 patients, selected for their HLA-A02 and/or -A24 alleles. Most of the cancer peptide-specific TILs expressed a Th1 profile and cytotoxic activity against target cells. The effector functions of cancer peptide-specific T-cells obtained from the peripheral blood of the same patients were also studied, and the majority of peripheral blood peptide-specific T cells also expressed the Th1 functional profile. 

In conclusion, in most of patients with gastric adenocarcinoma, a specific type-1 T-cell response to GC antigens was detectable and would have the potential of hamper tumor cell growth. However, in order to get tumor cell killing *in vivo*, the activity and the number of cancer peptide-specific Th1 cells probably need to be enhanced by vaccination with the appropriate cancer antigenic peptides or by injection of the autologous tumor peptide-specific T cells expanded *in vitro*. These studies have laid the groundwork for a possible vaccination of gastric adenocarcinoma patients with specific peptides of tumor-associated antigens able to raise an effective immune response to gastric cancer.

## 4. Colorectal Cancer

### 4.1. Tumour-Infiltrating T-Cell Subsets in Colorectal Cancer

In recent years, different studies demonstrated the presence of T cell into neoplastic tissue of colorectal patients and also that the type, location, and density of tumor-infiltrating immune cells are of strong predictive impact influencing the behavior of human CRC [85, 86]. Although the exact mechanism remains uncertain, the adaptive immune system plays an important role in suppressing tumour progression [87, 88]. In the Table 1 we resumed the major studies correlating the TIL subsets and survival of CRC patients.

From the above, the tumour-infiltrating T cells may be at the same time, an indicator of the host immune response versus cancer cells and an attractive target for immunotherapy [18, 89, 90]. 

The TILs may also reflect specific molecular alterations associated with indolent tumour behaviour. Previous studies have shown that lymphocytic infiltration is associated with microsatellite instability (MSI) in colorectal cancer [91–93]. Truncated peptides produced by frameshift mutations due to MSI may be immunogenic and contribute to the host immune response [88, 89, 94]. However, at the time, very little is known about the interrelationship between TILs, MSI, and other tumour molecular features, such as the CpG island methylator phenotype (CIMP), global DNA hypomethylation, and KRAS, BRAF, and PIK3CA mutations.

Previous studies have reported that MSI [95], CIMP [96], BRAF mutation [97], *PIK3CA *mutation [98], and tumour LINE-1 hypomethylation [99] are associated with prognosis and that lymphocytic infiltration is associated with many of these molecular variables [92]. As such, to define the prognostic effect of tumour-infiltrating T cells independently of those potential confounders, large studies of colorectal cancers with extensive molecular characterization are needed. Most recently, Nosho and coll. [100], using a database of 768 colorectal cancers, analyzed the subsets of TILs in relation with molecular changes in patients with CRC. They demonstrated that the densities of CD8^+^, CD45RO^+^, and FOXP3^+^ cells were significantly associated with patient survival in univariate analyses (*P* trend *< *0.007). In the multivariate model, tumour-infiltrating CD45RO^+^-cell density, but not CD3^+^, CD8^+^, or FOXP3^+^-cell density, was significantly associated with survival (*P* = 0.0032). In multivariate linear regression analysis, MSI-high (*P* < 0.0001) and high-level tumour LINE-1 methylation (*P* = 0.0013) were independently associated with higher CD45RO^+^-cell density. The survival benefit associated with CD45RO^+^ cells was independent of MSI and LINE-1 status. In conclusion, tumour-infiltrating CD45RO^+^-cell density is a prognostic biomarker associated with longer survival of colorectal cancer patients, independent of clinical, pathological, and molecular features. In addition, MSI-high and tumour LINE-1 methylation level are independent predictors of CD45RO^+^-cell density. These results offer a possible mechanism by which MSI confers an improved clinical outcome and support efforts to augment the host immune response in the cancer microenvironment as a strategy of targeted immunotherapy.

As with all tumors analyzed so far, even for the CRC it is very important to evaluate the impact of Tregs on the specific immune responses against tumor-associated antigens (TAAs). The grade of local infiltration did not correlate with responses against well-defined TAAs as EpCAM, Her-2/neu, and CEA [101]. Depleting Tregs in PBMCs from CRC patients dramatically boosted the IFN-*γ* and TNF-*α* production in T cells, which were stimulated with a CEA peptide [102]. In spite of the unmasking of responses in opposition to other TAAs, recall antigens such as PPD were not affected suggesting a TAA-specific rather than a systemic immune suppression [103]. 

In an extremely ample analysis various TAA-specific Tregs were exclusively identified in CRC patients. Peptides for CEA, telomerase, Her-2/neu, and MUC-1 all led to an activation of Tregs [104]. TAA-specific Tregs were successfully identified using a p53 peptide [105]; in addition to CD4^+^ Tregs also CD8^+^CD28^−^ Tregs could be isolated from peripheral blood, tumor tissue, and metastatic lymph nodes of CRC patients [106]. These cells suppressed T cells in an IL-10-dependent fashion and were mainly CD194^+^, which may have contributed to their accumulation *via *recruitment. A recent study identified circulating and tumor-infiltrating CD28^+^CD8^+^ Tregs with a CD25^+^, FOXP3^+^, CD152^+^, GITR^+^, CD194^+^, TGF-*β* ^+^, and CD127^−^ phenotype [107]. Remarkably this type of Tregs was found in 90% of the CRC specimens but was totally absent in normal colonic tissue suggesting a cancer-specific presence without contribution to the physiologic epithelial homeostasis [108]. Ligands for CD194 (e.g., CCL17 or CCL22) were in contrast to IL-6 and TGF-*β* not highly expressed in the tumor tissue, altogether indicating a conversion from CD8^+^ rather than a tumor-directed migration as the cause for the observed infiltration. In another recent study CXCL11 produced by CRC-derived CD68^+^ myeloid cells is suggested to be a promising chemoattractant for Tregs [109].

### 4.2. T-Cell-Based Immunotherapy in CRC Patients

T-cell-based immunotherapy (TCI) was first described in 1988 [110], but the decisive improvement in efficacy came in 2002 with the introduction of an immunodepleting preparative regimen given before the adoptive transfer, which could result in the clonal repopulation of patients with antitumour T cells [111]. Of patients with metastatic melanoma refractory to all other treatments, 50% will experience an objective response, some with complete responses [112]. Responses can be durable and are seen in all organ sites, including the brain. Recent studies demonstrating that normal human T cell can be genetically engineered to recognize cancer antigens and mediate cancer regression *in vivo *have opened opportunities for enhancing and extending the TCI approach to patients with a wide variety of cancer types [113]. These studies provide a valuable guide to the immunological principles that form the basis of effective immunotherapy for CRC patients.

Most nonhematopoietic tumors such as CRC express MHC class I molecules, but do not express MHC class II molecules, therefore it is believed that the predominant tumor-specific cell-mediated immune effector mechanism is the killing by CTL. However, the clinical history of the patient with cancer often demonstrates the failure of the immune system to eliminate the tumor [114]. It is now generally accepted that this is mostly due to poor tumor-specific MHC class II-restricted CD4^+^ T helper generated in tumor-bearing hosts [115–117] and that Th cells are required for priming and clonal expansion of specific CTL following reencounter with antigen [118–121].

Although at clinical level, TCI results are still preliminary [122], nevertheless the importance of including CD4^+^ together with CD8^+^ T cells to induce optimal therapeutic effects has been established [112, 123].

For this purpose and to optimize the antitumor immunological arms in terms of specificity and long-lasting memory, vaccination with tumor cells transduced with the AIR-1-encoded CIITA, the MHC class II [MHC-II] gene transactivator [124, 125], has been explored with the idea that CIITA-transfected cells may act as “surrogate APC” for optimal triggering of tumor-specific Th cells and thus facilitate the recognition of TAA presented by tumor cell MHC-II molecules. Indeed, the group of Accola showed that complete rejection and long-lasting antitumor memory could be obtained after vaccination with CIITA-expressing TS/A mammary adenocarcinoma [126–128]; Most recently, the same group [129] demonstrated that CIITA-expressing C51 colon adenocarcinoma cells are rejected in high percentage of mice or strongly reduced in growth. Induction of antitumor immunity depended on the ability of the MHC-II-positive tumor cells to trigger CD4^+^ T cells, which in turn induce stimulation and maturation of CTL effectors. Importantly, they showed that immune CD4^+^ Th cells can induce protective antitumor responses in naive mice injected with parental nontransfected tumor cells. Purified CD4^+^ T cells from C51-CIITA vaccinated and challenged mice were also efficacious in preventing tumor growth of C51 tumor, as 50% of the animals were protected and the remaining 50% displayed a significant growth retardation. Similar results were obtained when immune CD8^+^ T cells were used in adoptive transfer, even if CD4^+^ T cells were clearly superior to CD8^+^ T cells in antitumor protective function. Interestingly, the protective phenotype was associated to both a Th1 and Th2 polarization of the immune effectors. 

In conclusion, these results demonstrated that tumor cell modification by CIITA may offer an alternative strategy not only for preventive vaccination but also for the generation of more efficacious TCI for CRC patients.

In recent years it has also become increasingly the cancer stem cell theory [130], the idea that cancers are composed of several types of cells, and that only a small population of cancer cells that can regenerate cancer tissues, much as normal tissue can be regenerated only by a small population of stem-like cells. Recently, cancer stem-like cells and tumor-initiating cells (CSCs/TICs) have been isolated from various types of malignancies, including colon cancer [131–135]. 

In colon cancer, CSCs/TICs can reinitiate tumors that resemble mother colon cancer tissues morphologically when transplanted into immunodeficient mice [132]. Furthermore, these CSCs/TICs have higher tumorigenic potential than do non-CSCs/TICs, suggesting that they are essential for tumor maintenance and distant metastasis [132].

 Previous reports have shown that CSCs/TICs are resistant to a variety of treatments, including chemotherapy and radiotherapy, with varied mechanisms of resistance, including high expression of drug transporters, relative cell cycle quiescence, high levels of DNA repair machinery, and resistance to apoptosis [136].

In recent times Inoda and coll. shown that CTL specific for the tumor-associated antigen CEP55 can efficiently recognize colon CSCs/TICs both *in vitro *and *in vivo*. The authors isolated CSCs/TICs as side population (SP) cells from colon cancer cell lines SW480, HT29, and HCT15. The SP cells expressed high levels of the stem cell markers SOX2, POU5F1, LGR5, and ALDH1A1 and shown resistance to chemotherapeutic agents (irinotecan or etoposide). To evaluate the susceptibility of SP cells to CTLs, they used CTL clone 41, which is specific for the CEP55-derived antigenic peptide Cep55/c10orf3_193 [137, 138]. The SP cells expressed HLA class I and CEP55 at the same level as the main population cells. The SP cells were susceptible to CTL clone 41 at the same level as main population cells. Furthermore, adoptive transfer of CTL clone 41 inhibited tumor growth of SW480 SP cells *in vivo*. 

These results suggest that Cep55/c10orf3_193 [137, 138] peptide-based cancer vaccine therapy or adoptive cell transfer of the CTL clone is a possible approach for targeting chemotherapy-resistant colon CSCs/TICs.

## 5. Conclusion 

Despite advances in clinical diagnostics, surgical techniques, and development of new chemo/radiotherapy regimens the prognosis of gastrointestinal oncology remains poor, and the need for new treatment options, such as immunotherapy, is imperative.

Studies of T-cell-based immunotherapy have clearly demonstrated that the administration of highly avid anti-tumour T cells directed against a defined target can mediate the regression of large, vascularized, metastatic cancers and provide guiding principles as well as encouragement for the further development of adoptive T-cell therapy for cancer patients.

In this paper we have reported the evidence of the key role of T-cell response versus cancer of the digestive system and the results obtained in different clinical trials using T-cell immunotherapy. 

We showed that for pancreatic cancer as well as for both gastric and colorectal cancer good results were obtained in some clinical settings but in order that T-cell-based immunotherapy become a real treatment for gastrointestinal oncology, several problems must be solved.

A major problem with the application of TCI is that it is a highly personalized treatment and does not easily fit into current modes of oncological practice. The treatment is expensive, labour-intensive, and requires high laboratory expertise. In essence, a new reagent needs to be created for each patient, and this patient-specific nature of the treatment makes it difficult to commercialize. 

Moreover, currently the major challenge in the field is to conduct randomized clinical trials demonstrating sufficient clinical benefit to justify the logistics and costs of customized cellular therapies. In many clinical trials, patients are enrolled at an advanced cancer stage, and this aspect could determine an unfavourable outcome; thus, it would be very interesting to plan clinical trials in early-stage of cancer because it would be possible that gastrointestinal cancer immunotherapeutic approaches confer a survival advantage when applied earlier during the course of the disease, such as in the adjuvant setting. 

However, the big hurdle to make immunotherapy approach successful for gastrointestinal oncology remains the immune evasion strategies set up by the tumor resulting in avoidance of both innate and adaptive immunity.

Investigations during the past few years have provided new insights into the cellular and molecular mechanisms involved in the bidirectional crosstalk between cancer cells and the immune cells. Understanding this functional dialogue and the hierarchical status of different tumor-immune escape stratagems at different stages of tumor development will guide the design of novel therapeutic strategies aiming to demolish the “tumor fortress”.

Thus, it will be of particular interest to study the kinetics of the interactions between different inhibitory molecules and endogenous factors that influence the expansion and trafficking of Tregs and tolerogenic DCs within tumor-draining lymph nodes and the tumor surroundings.

On the basis of clinical and experimental evidence, it is reasonable to conclude that successful therapy for gastrointestinal oncology must involve a combination approach, which should involve systemic chemotherapy and transplantation to reduce the burden or to eliminate immune suppressive cells, together with tailor-made immunotherapies customized to each single patient. 

## Figures and Tables

**Table 1:** Studies correlating colorectal cancer patient survival with TIL subsets.

N° of events				High density of T-cells (versus low density)				References
Sample size	OS	CS	Disease stage	T-cell subset analysed	5-year CS, OS, or DFS, long-rank *P* value	CS, OS, or DFS univariate HR (95% Cl)*, *P* value	CS, OS, or DFS multivariate HR (95% Cl)*, *P* value	
131	—	—	Dukes' A–D	CD8^+^	*P* = 0.0003 (OS)	—	0.61 (0.41–0,89)	[139]; 58: 3491–3494

				CD3^+^	*P* = 0.004 (OS)	—	*P* = 0.011 (OS) 0.40 (0.19–0.85) (OS)	
109	37	—	II-III	CD8^+^	*P* = 0.0008 (OS)	—	0.33 (0.15–0.73) (OS)	[140]; 159: 297–304
				GZMB^+^	*P* = 0.0001 (OS)	—	0.23 (0.10–0.50) (OS)	

				CD8^+^	77% (versus 38%) *P* = 0.011 (CS)	—	—	
93	59	47	Dukes' C	CD45RO^+^	66% (versus 33%) *P* = 0.002 (CS)	—	—	[141]; 17: 25–29
				CD68^+^	60% (versus 37%) *P* = 0.033 (CS)	—	—	

72	—	—	I–IV	CD134^+^	*P* = 0.02 (CS)	—	—	[142]; 183: 512–518

41	25	—	Dukes' A–D	CD4^+^/CD8^+^ ratio	22% (versus 61%) (OS)	—	*P* = 0.046 (OS)	[143]; 52: 423–428

97	—	—	—	CD8^+^	—	*P* = 0.01 (OS)	—	[144]; 10: 309–313.

152	—	—	III	CD8^+^	*P* < 0.001 (CS)	—	—	[145]; 35:808–816

93	—	—	II-III	CD8^+^	*P* = 0.03 (DFS)	—	0.56 (0.32–0.99) *P* = 0.04 (DFS)	[146]; 84: 493–501

371	—	74	I–IV	CD8^+^	*P* < 0.0001 (CS)	—	0.43 (0.23–0.83) *P* = 0.01 (CS)	[147]; 91: 1711–1717

336	158	—	Dukes' A–D	CD45RO^+^	65% (versus 35%) *P* < 0.0001 (OS) 72% (versus 50%) *P* < 0.001 (DFS)	—	*P* = 0.02 (OS)	[148]; 353: 2654–2666

				CD3^+^	*P* < 0.05 (CS)	—	—	
117	—	—	Dukes' A–D	CD8^+^	*P* < 0.25 (CS)	—	—	[149]; 4: 1351–1357
				CD16^+^	*P* < 0.04 (CS)	—	—	

				CD3^+^	73% (versus 40%) *P* < 0.0001 (OS) 81% (versus 54%) *P* = 0.0012 (DFS)	—	0.53 (0.40–0.70) *P* < 0.0001 (OS) 0.42 (0.29–0.60) *P* < 0.0001 (DFS)	
				CD8^+^	72% (versus 50%) *P* < 0.0001 (OS)	—	—	
406	—	—	I–III	CD45RO^+^	82% (versus 56%) *P* = 0.0002 (DFS) 68% (versus 33%) *P* < 0.0003 (OS)	—	—	[150]; 313: 1960–1964
				GZMB^+^	77% (versus 37%) *P* = 0.002 (DFS) 72% (versus 61%) *P* = 0.15 (OS)	—	—	
				CD3^+^, CD45RO^+^	84% (versus 68%) *P* = 0.39 (DFS) *P* < 0.0001 (DFS)	—	—	

587	—	—	I-II (MSS only)	CD8^+^	—	0.47 (0.33 0.68) *P* < 0.001 (CS)	0.47 (0.30–0.73) *P* = 0.001 (CS)	[151]; 112: 495–502

392	—	226	I-II (Rectum only)	CD8^+^	—	0.55 (0.41–0.74) *P* < 0.001 (CS)	0.63 (0.45–0.88) *P* = 0.006 (CS)	[152]; 99: 1712–1717

101	—	—	II-III	CD3^+^/FOXP3^+^ ratio	71% (versus 62%) (OS) 67% (versus 46%) (DFS)	0.57 (0.30–1.09) *P* = 0.087 (OS) 0.46 (0.24–0.90) *P* = 0.021 (DFS)	0.47 (0.24–0.94) *P* = 0.039 (DFS)	[153]; 137: 1270–1279

					Node-negative *P* = 0.01 (CS)			
286	—	136	II-III	CD3^+^	Node-positive *P* = 0.66 (CS)	—	—	[154]; 10: 877–884
					Node-negative *P* = 0.006 (DFS)			
					Node-positive *P* = 0.62 (DFS)			

					MSS group 162% (versus 46%) *P* = 0.004 (CS)	—	0.73 (0.60–0.90) *P* = 0.019 (CS)	
1232	—	—	I-III	FOXP3^+^	MSS group 2 (CS) 60% (versus 44%) *P* = 0.001 (CS)	—	0.70 (0.60–0.90) *P* = 0.007 (CS)	[155]; 126: 2635–2643
					MSI group (CS) 75%? (versus 63%?) *P* = 0.029 (CS)	—	0.63 (0.3–1.2) *P* = 0.13 (CS)	

				CD8^+^	—	0.74 (0.67–0.82) *P* < 0.0001 (CS)	NS (CS)	
445	—	—	II-III	CD45RO^+^	—	0.74 (0.65–0.84) *P* < 0.0001 (CS)	NS (CS)	[156]; 27: 186–192
				FOXP3^+^	—	0.78 (0.70–0.87) *P* < 0.0001 (CS)	0.54 (0.38–0.77) *P* = 0.001 (CS)	

				CD8^+^	*P* < 0.0001 (OS and DFS)	—	—	
411	—	—	I-II	CD45RO^+^	*P* < 0.0001 (OS and DFS)	—	—	[157]; 27: 5944–5951
				CD8^+^ plus CD45RO^+^	*P* < 0.0001 (OS and DFS)	—	*P* < 0.0001 (CS, OS and DFS)	

				CD3^+^	*P* = 0.04 (OS)	—	0.54 (0.18–1.59) *P* = 0.26 (OS)	
209	100	100	I–IV	CD8^+^	*P* = 0.04 (OS)	—	2.06 (0.67–6.39) *P* = 0.21 (OS)	[158]; 11: 19
				GZMB^+^	—	—	1.18 (0.45–3.13) *P* = 0.74 (OS)	

94	—	—	I–IV	CD8^+^/FOXP3^+^ ratio	*P* = 0.01 (OS)	0.35 (0.15–0.81) *P* = 0.014 (OS)	0.40 (0.17–0.94) *P* = 0.035 (OS)	[159]; 59: 653–661

57	—	—	—	FOXP3^+^	—	*P* = 0.0009 (DFS)*P* = 0.0005 (OS)	—	[160]; 33: 435–441

				CD3^+^	*P* = 0.010 (DFS) *P* = 0.061 (OS)	*P* = 0.003 (DFS) *P* = 0.039 (OS)	0.20 (0.02–2.60) *P* = 0.22 (DFS)	
87	—	—	II	CD25^+^	*P* = 0.013 (DFS) *P* = 0.15 (OS)	*P* = 0.002 (DFS) *P* = 0.017 (OS)	0.22 (0.02–2.35) *P* = 0.21 (DFS)	[161]; 116:5188–5199
				CD45RO^+^	*P* = 0.049 (DFS) *P* = 0.16 (OS)	*P* = 0.014 (DFS) *P* = 0.037 (OS)	0.24 (0.02–1.10) *P* = 0.014 (DFS)	
				FOXP3^+^	*P* = 0.009 (DFS) *P* = 0.027 (OS)	*P* = 0.005 (DFS) *P* = 0.040 (OS)	0.14 (0.07–0.85) *P* = 0.027 (DFS)	

				CD3^+^	79% (versus 75%) Q4 (versus Q1)^†^ *P* = 0.19 (CS)	0.73 (0.49–1.08) Q4 (versus Q1)^†^ *P* = 0.070^‡^ (CS)	1.30 (0.81–2.07) Q4 (versus Q1)^†^ *P* = 0.16^‡^ (CS)	
768	366	229	I-IV	CD8^+^	78% (versus 66%) Q4 (versus Q1)^†^ *P* = 0.026 (CS)	0.61 (0.42–0.88) Q4 (versus Q1)^†^ *P* = 0.007^‡^ (CS)	0.81 (0.52–1.27) Q4 (versus Q1)^†^ *P* = 0.34^‡^ (CS)	[162]; 222: 350–366
				CD45RO^+^	83% (versus 68%) Q4 (versus Q1)^†^ *P*< 0.0001 (CS)	0.40 (0.26–0.60) Q4 (versus Q1)^†^ *P*< 0.0001^‡^ (CS)	0.51 (0.32–0.80) Q4 (versus Q1)^†^ *P* = 0.034^‡^ (CS)	
				FOXP3^+^	80% (versus 64%) Q4 (versus Q1)^†^ *P*< 0.0001 (CS)	0.48 (0.32–0.70) Q4 (versus Q1)^†^ *P*< 0.0001^‡^ (CS)	0.89 (0.59–1.34) Q4 (versus Q1)^†^ *P* = 0.034^‡^ (CS)	

*HR is based on comparing high versus low score of a given T-cell subset. ^†^Quartile of density (Q1-4, first to fourth quartile). ^‡^ *P* for trend. Cl: confidence interval; CS: colorectal cancer-specific survival; DFS: disease-free survival; HR: hazard ratio; MSI: microsatellite instability; NS: not significant; OS: overall survival.
